# Investigating the Role of Guanosine on Human Neuroblastoma Cell Differentiation and the Underlying Molecular Mechanisms

**DOI:** 10.3389/fphar.2021.658806

**Published:** 2021-04-27

**Authors:** Natale Belluardo, Giuseppa Mudò, Valentina Di Liberto, Monica Frinchi, Daniele F. Condorelli, Ugo Traversa, Francisco Ciruela, Renata Ciccarelli, Patrizia Di Iorio, Patricia Giuliani

**Affiliations:** ^1^ Department of Biomedicine, Neuroscience and Advanced Diagnostic, University of Palermo, Palermo, Italy; ^2^ Department of Biomedical and Biotechnological Sciences, Section of Medical Biochemistry, University of Catania, Catania, Italy; ^3^ Department of Life Sciences, University of Trieste, Trieste, Italy; ^4^ Pharmacology Unit, Department of Pathology and Experimental Therapeutics, Faculty of Medicine and Health Sciences, Institute of Neurosciences, University of Barcelona, L’Hospitalet de Llobregat, Spain; ^5^ Neuropharmacology and Pain Group, Neuroscience Program, Institut d’Investigació Biomèdica de Bellvitge, IDIBELL, L’Hospitalet de Llobregat, Spain; ^6^ Department of Medical, Oral and Biotechnological Sciences, “G. D’Annunzio” University of Chieti-Pescara, Chieti, Italy; ^7^ Center for Advanced Studies and Technology, CAST, “G. D’Annunzio” University Foundation, Chieti, Italy

**Keywords:** SH-SY5Ydifferentiation, neuroblastoma, guanosine, purine nucleoside phosphorylase, guanine, protein kinase C, guanylate cyclase, heme oxygenase

## Abstract

Neuroblastoma arises from neural crest cell precursors failing to complete the process of differentiation. Thus, agents helping tumor cells to differentiate into normal cells can represent a valid therapeutic strategy. Here, we evaluated whether guanosine (GUO), a natural purine nucleoside, which is able to induce differentiation of many cell types, may cause the differentiation of human neuroblastoma SH-SY5Y cells and the molecular mechanisms involved. We found that GUO, added to the cell culture medium, promoted neuron-like cell differentiation in a time- and concentration-dependent manner. This effect was mainly due to an extracellular GUO action since nucleoside transporter inhibitors reduced but not abolished it. Importantly, GUO-mediated neuron-like cell differentiation was independent of adenosine receptor activation as it was not altered by the blockade of these receptors. Noteworthy, the neuritogenic activity of GUO was not affected by blocking the phosphoinositide 3-kinase pathway, while it was reduced by inhibitors of protein kinase C or soluble guanylate cyclase. Furthermore, the inhibitor of the enzyme heme oxygenase-1 but not that of nitric oxide synthase reduced GUO-induced neurite outgrowth. Interestingly, we found that GUO was largely metabolized into guanine by the purine nucleoside phosphorylase (PNP) enzyme released from cells. Taken together, our results suggest that GUO, promoting neuroblastoma cell differentiation, may represent a potential therapeutic agent; however, due to its spontaneous extracellular metabolism, the role played by the GUO-PNP-guanine system needs to be further investigated.

## Introduction

Neuroblastoma (NB) is the most common extracranial solid tumor of childhood accounting for approximately 10% of all pediatric cancers, with most cases diagnosed before 5 years of age. NB prognosis and clinical course are extremely variable and depend on the patient’s age, tumor stage, and location. Thus, patients can be classified into three pretreatment risk groups (low, intermediate, and high risk) with different outcomes ranging from “very good” in the low-risk group, with the possibility of spontaneous regression, to “poor” outcome in the high-risk group with reduced chances of survival (Whittle et al., 2017; Newman et al., 2019).

Treatment is tailored according to the risk assignment. A very intensive approach is used for high-risk patients, with treatment options including chemotherapy, surgical resection, high-dose chemotherapy with autologous stem cell rescue, radiotherapy, immunotherapy, and differentiating therapy (Smith and Foster, 2018). Interestingly, this last approach is based on the knowledge that NB derives from neural crest cell precursors failing to differentiate, thus remaining blocked at an undifferentiated stage. Therefore, agents able to induce cell differentiation are an attractive therapeutic approach. However, few agents are available to this aim, and retinoic acid (RA) is the most commonly used but, unfortunately, resistance to this agent is frequent (Reynolds et al., 2003). This highlights the need of developing new potential differentiating agents.

Guanine-based purines are a group of naturally occurring purines including guanosine mono-, di-, and tri-phosphate nucleotides (GMP, GDP, and GTP, respectively), the nucleoside guanosine (GUO), and the nucleobase guanine (GUA). In addition to well-known intracellular roles (i.e., regulation of G-protein activity linked to metabotropic receptors; formation of the second messenger cyclic GMP–cGMP-), growing evidence pointed out important extracellular effects of GTP and GUO, suggesting that they maybe considered as neuromodulatory signaling agents regulating many different physiological functions at both the central and peripheral nervous system (Di Liberto et al., 2016; Tasca et al., 2018). Among these, it is potentially relevant to NB management the GUO capacity to induce the differentiation of several cell types, as demonstrated by *in vivo* and *in vitro* studies. Indeed, GUO treatment stimulated neurogenesis in a rat model of Parkinson’s disease (Su et al., 2009), and its chronic administration increased differentiated neurons in mouse hippocampal dentate gyrus (Bettio et al., 2016). Again, in hippocampal, cerebellar, and pheochromocytoma (PC12) cell cultures, GUO stimulated neurite outgrowth, and in PC12 cells, this effect was enhanced by the copresence of nerve growth factor (Gysbers and Rathbone, 1996; Böcklinger et al., 2004; Bau et al., 2005). GUO also promoted B16F10 melanoma cell differentiation and inhibited cell motility, leading to a decreased melanoma malignancy (Naliwaiko et al., 2008). Noteworthy, Guarnieri et al. (2009) reported that extracellular GUO induced a mature neuronal phenotype in NB cells, but the signaling pathways involved have not been investigated.

Altogether, these findings represented the natural premise that prompted us to further investigate the differentiation effect of GUO on a human NB cell line, SH-SY5Y, trying to identify some pathways involved in this potential effect. In parallel, we evaluated the activity of purine nucleoside phosphorylase (PNP), the enzyme involved in GUO conversion into GUA, since it is expressed in almost all tissues (Moriwaki et al., 1999) and might deeply affect the activity of GUO on cells. In our opinion, results obtained in this study could open the way to identify new therapeutic agents to implement the differentiation-based therapies in NB.

## Materials and Methods

### Materials and Chemicals

The human SH-SY5Y cell line was purchased from European Collection of Authenticated Cell Cultures (Salisbury, United Kingdom); NG-nitro-L-arginine methyl ester (L-NAME), GF109203X, dipyridamole, and LY294002 from Tocris (Milan, Italy); and all other drugs, antibodies, and reagents were obtained from Sigma-Aldrich unless otherwise stated.

### Cell Culture and Treatment

SH-SY5Y cells were cultured in Dulbecco’s Modified Eagle Medium (DMEM)/F12 medium supplemented with 2 mM L-glutamine and 1% penicillin/streptomycin and different concentrations of inactive fetal bovine serum (FBS, 0–10%) and maintained in a humidified atmosphere of 5% CO_2_ at 37°C. For evaluating the neuritogenic effect of GUO, cells were treated with various concentrations (GUO 1–300 µM) for different times (24–96 h). Medium containing GUO was changed every 48 h. When used, the inhibitors of nucleoside transporters, propentofylline 100 µM, S-(4-nitrobenzyl)-6-thioinosine (NBTI) 10 μM, and dipyridamole 10 μM were added to the medium 1 h before and during 48 h GUO treatment. In some experiments, cells were treated 30 min before and during all GUO treatment, with the A_1_ adenosine receptor antagonist (1,3-dipropyl-8-cyclopentylxanthine-DPCPX-100 nM), the A_2A_ receptor antagonist ([4-(2-[7-amino-2-{2-furyl}{1,2,4}triazolo{2,3a}{1,3,5}triazin-5-ylamino]ethylphenol]-ZM241385–50 nM), or with the selective inhibitors of 1) phosphatidyl inositol-3-kinase (PI3K) (LY294002, 25 µM), 2) protein kinase C (PKC) (GF109203X, 1 µM), 3) soluble guanylate cyclase (sGC) (oxadiazolo[4,3-a]quinoxalin-1-one–OQD-, 10 µM), 4) nitric oxide synthase (NOS) (L-NAME, 5 µM), or 5) heme oxygenase (HO) (zinc protoporphyrin IX -ZnPP-1 µM).

### Neurite Outgrowth Assay

To evaluate the effect of test compounds on neurite outgrowth, at the end of each experiment, cells were photographed using an Axiocam MRC 5 camera connected to an upright Zeiss Axiovert 200 microscope equipped with an objective 20X Plan-Apo/0.75 NA, total magnification 200x (Zeiss, Jena, Germany). Pictures were taken from 3 to 5 independent microscopic fields from at least 3 independent experiments under blind conditions. Images were acquired using the AxioVision software in bright field settings (Zeiss). For each experiment, at least 100 cells were counted and the number of neuron-like differentiated cells, defined as cells with one or more neurites longer than the diameter of the cell body, was determined to evaluate the percentage of neurite-bearing cells.

### Immunocytochemistry

SH-SY5Y cells were seeded in a six-well culture plate containing rectangular glass coverslips and exposed to different substances. Briefly, at the indicated time, cells were fixed in 2% paraformaldehyde for 30 min and washed with phosphate-buffered saline. Then, cells were permeabilized with Triton X-100 for 5 min and subsequently blocked with a solution containing 1% serum bovine albumin and 5% normal goat serum (1 h, 37°C). Cells were incubated at 4°C overnight with primary antibodies: mouse anti-β-tubulin antibody (1:250), mouse anti-MAP2 antibody (1:250), or mouse anti-NeuN antibody (1:100). Cells were then washed and stained with the secondary antibody (1 h, 37°C). Coverslips were rinsed and mounted with DABCO-glycerol. The observations were made with a fluorescence microscope Nikon Eclipse E800 and images acquired by a DMX 1200 photo camera.

### Measurement of Extracellular Guanosine and Guanine Levels and Enzyme Activity Assay

To evaluate the concentration of extracellular GUO and GUA, at the indicated time, an aliquot of the medium was taken and immediately heat-inactivated for 5 min at 70°C to prevent any further metabolic degradation. After centrifugation, the supernatant was filtered through 0.2 µm filters (Millipore, Vindrome, Italy) and analyzed by HPLC as reported below.

Samples containing PNP were obtained as described by Giuliani et al. (2017). Briefly, SH-SY5Y cells were incubated in serum-free medium without or with 100 µM GUO. After 6 and 24 h, the medium was taken and the enzyme present was concentrated using Amicon Ultra 2 ml filters (cutoff 10 K, Merck Millipore), while cells were scraped in lysis buffer (5 mM HEPES pH 8.5, 2 mM EDTA, and protease inhibitor cocktail) and sonicated to obtain cytosolic extracts. Protein content was quantified using a colorimetric protein assay kit (Bio-Rad, Segrate, Italy). PNP activity was evaluated by measuring the transformation of GUO, the enzyme’s substrate, into GUA by HPLC analysis as previously reported (Giuliani et al., 2016). Briefly, the enzymatic reaction occurs in HEPES (50 mM; pH 7.0) containing 50 mM inorganic phosphate plus an aliquot of the concentrated medium or a cytosolic sample as a source of PNP. 100 µM GUO was then added and the mixture was incubated by shaking at 37°C for 15 min. In the experiments aimed to evaluate the specificity of the enzyme assay, the PNP inhibitor, forodesine 1 µM (D.B.A, Segrate, Italy), was added to the reaction mixture before GUO. The reaction was stopped by heating the mixture at 70°C for 5 min. After centrifugation, the supernatant was filtrated before HPLC analysis.

The HPLC (Agilent 1,100 Series, Waldbronn, Germany) was equipped with a thermostated column compartment, a diode array detector, and a fluorescence detector. The separation was achieved by a Phenomenex Kinetex pentafluorophenyl analytical column (Phenomenex INC., Bologna, Italy) kept at 35°C and applying a 15-min nonlinear gradient with a flow rate of 1 ml/min (for further details see Giuliani et al., 2016). The fluorescents GUO and GUA were monitored at an excitation wavelength of 260 nm and an emission wavelength of 375 nm. PNP activity was expressed as milli-international units (mIU) being 1 IU of enzyme the amount of PNP that catalyzes the conversion of 1 μmol of substrate per min.

### Statistical Analysis

Data were analyzed by one-way or two-way ANOVA followed by Tukey’s or Dunnett’s post hoc test for multiple comparisons using GraphPad Prism software. All data were expressed as mean ± SEM of at least three independent experiments in duplicate or triplicate. *p* values <0.05 were considered statistically significant.

## Results

### Guanosine Induces Differentiation of Human Neuroblastoma SH-SY5Y Cells

Although serum present in the culture medium provides optimal conditions for cell growth, it can interfere with the differentiation process, thus differentiation is usually performed by reducing serum concentrations (Brunner et al., 2010; Magalingam et al., 2020). However, serum deprivation can cause cell death (Braun et al., 2011; Rashid and Coombs, 2019). Therefore, we first evaluated the lowest serum concentration that did not affect SH-SY5Y cell survival. To this aim, cell viability was assessed by MTT assay in cultures incubated for 24 h up to 96 h with a medium containing different serum concentrations (0–10%). As detailed in the Supplements, either serum-free medium or medium containing 0.5% serum decreased cellular viability after 48 h, while the presence of 1% FBS did not modify it. Conversely, medium containing 5 or 10% FBS caused a time- and concentration-dependent proliferative effect (Supplementary Figure S1). Thus, we performed the subsequent experiments using a culture medium containing 1% serum.

**FIGURE 1:**
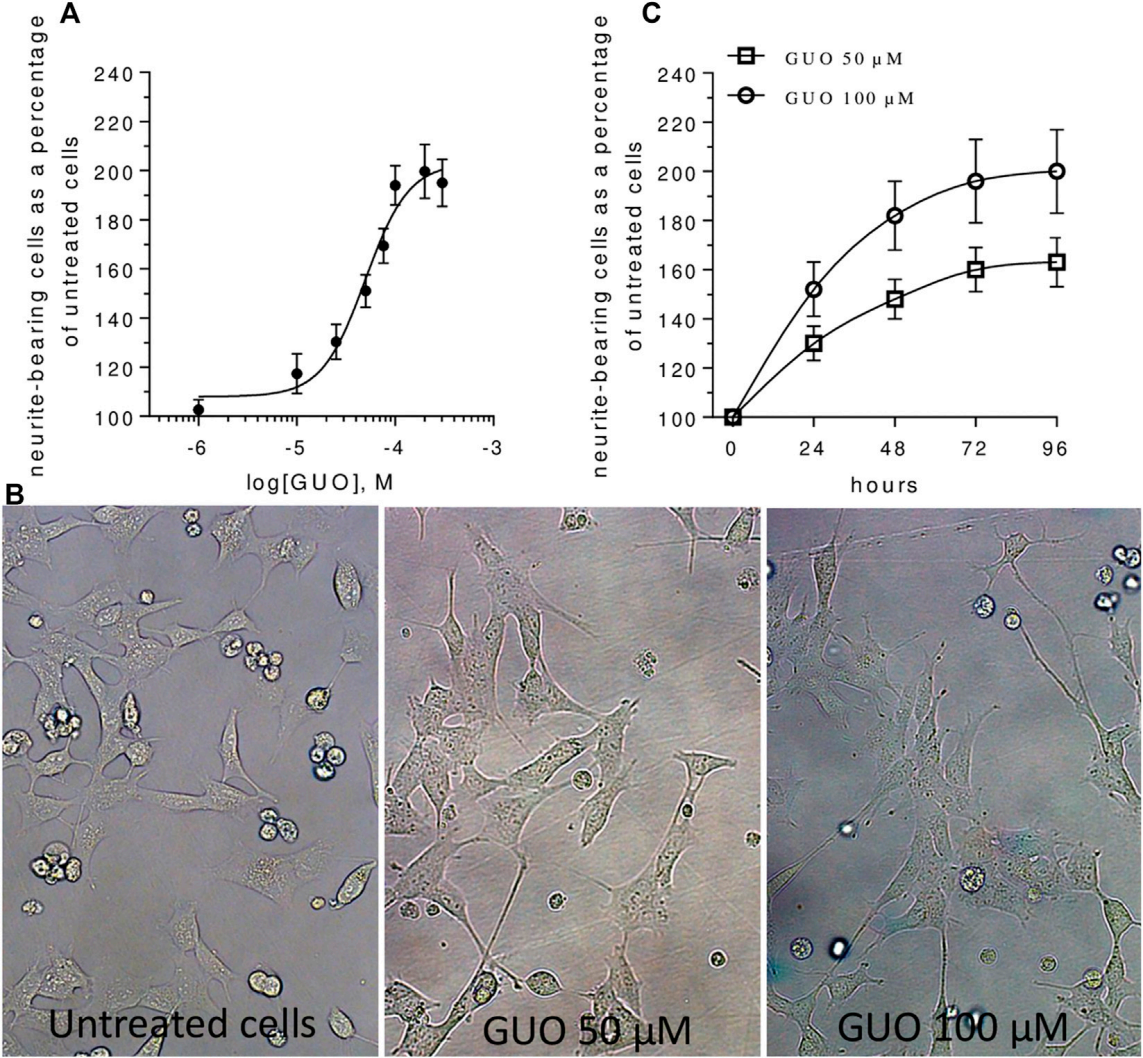
Guanosine induces SH-SY5Y neuroblastoma cell differentiation in a dose- and time-dependent manner. **(A)** For the concentration-response curve, increasing concentrations (1–300 µM) of GUO were added for 48 h in medium containing 1% FBS. A non-linear regression analysis was performed and Emax and EC50 were calculated. **(B)** Representative images of SH-SY5Y cells cultures treated with 0, 50, and 100 μM GUO in medium with 1% FBS after 48 h. **(C)** For the time-dependent curve, cell cultures were incubated with GUO (50–100 µM) for different times (0–96 h) in a medium culture containing 1% FBS. In all cases, the number of differentiated cells, defined as cells having neurites more than one cell body length, was determined using a upright Zeiss Axiovert 200 microscope, total magnification 200x (Zeiss, Jena, Germany). Values are expressed as a percentage of neurite-bearing cells vs untreated cells and represent the mean ± SEM from at least three to five independent experiments.

Then, we investigated the effect of increasing concentrations of GUO (1–300 µM) on NB cell differentiation by calculating the number of cells bearing neurites with a length major than the cell body. The GUO effect was first evaluated by exposing cells to 48 h treatment, a time previously reported as suitable for GUO to affect PC12 cell differentiation (Gysbers and Rathbone, 1992; Bau et al., 2005). In comparison to untreated cells, which showed few and very short neurite-like projections, GUO-treatment increased in a dose-dependent manner the number of neuron-like differentiated cells (Figures 1A,B). The maximum effect was reached at the concentration of 100 μM, whereas the calculated EC50 value was 49.67 ± 6.22 µM. Next, to appreciate the neuritogenic activity of GUO over time, we performed a time-course study using 50 and 100 µM GUO, able to cause half and maximal differentiation effects, respectively. In untreated cultures, the percentage of neuron-like cells was quite constant, varying from 10 to 20% throughout the observation period. As expected, differentiation was greater in cells treated with 100 µM GUO than in those exposed to 50 μM, although in both cases, the maximal effect was reached after 48–72 h treatment (Figure 1C).

**FIGURE 2:**
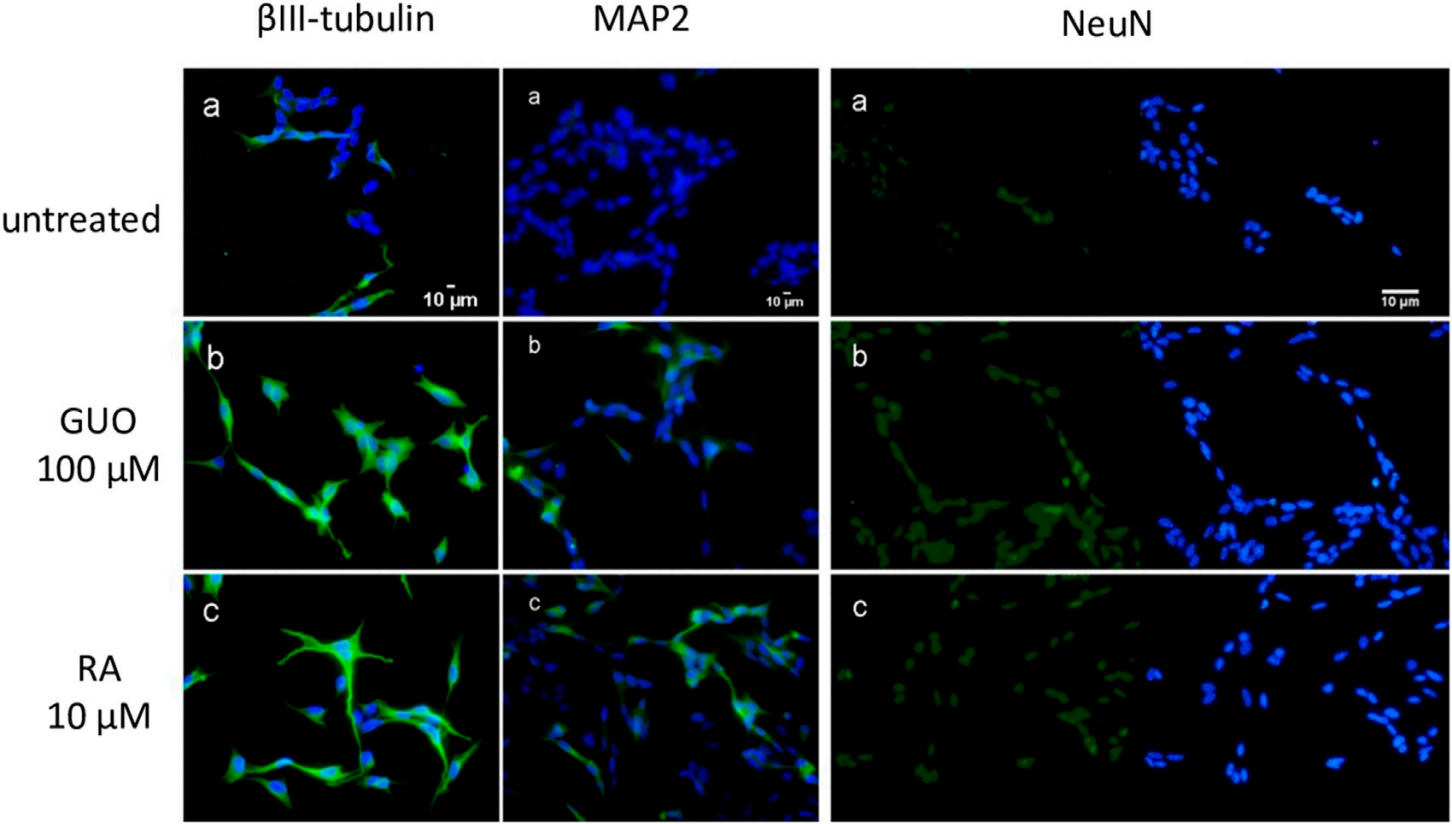
Guanosine increases the expression of specific mature neuronal markers in SH-SY5Y neuroblastoma cells. SH-SY5Y cells were cultured for 48 h without treatment **(a)** or in the presence of 100 µM GUO **(b)** or 10 µM RA **(c)**. βIII-Tubulin and MAP2 filaments are highlighted in green while nuclei are stained in blue with DAPI. For NeuN expression, on the left side of the image nuclei are stained in green with NeuN while on the right side of the image nuclei are stained in blue with DAPI. Images are representative of one of three independent experiments.

To further characterize SH-SY5Y cells cultured under our conditions, we also performed immunocytochemical staining for specific mature neuronal markers such as βIII tubulin, MAP2 (microtubule-associated protein 2), and NeuN, a nuclear neuron-specific marker (Soltani et al., 2005; Borsani et al., 2020). We also compared these results with those obtained with the most common and established differentiating agent, RA 10 µM (Kovalevich and Langford, 2013). We found that like RA 10 μM, Guo 100 µM for 48 h increased the expression of the neuronal markers related to the differentiation compared to untreated cells (Figure 2). Furthermore, by using phalloidin staining we monitored the change in F-actin reorganization and appreciate a time-dependent emersion and extension of neurites (Supplementary Figure S2).

### The Effect of Guanosine on SH-SY5Y Neuron-Like Cell Differentiation Involves Different Mechanisms

**FIGURE 3:**
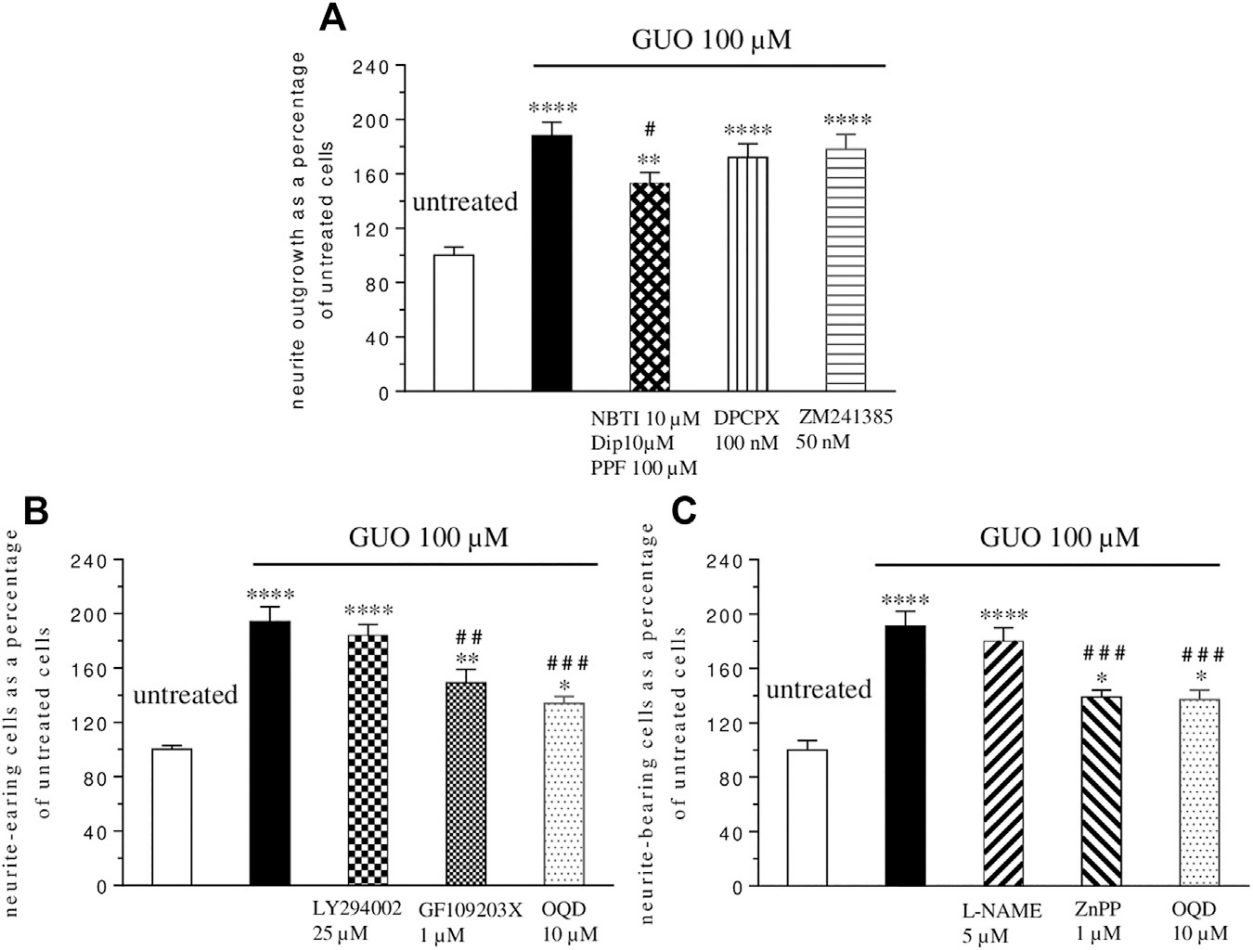
Mechanisms involved in guanosine-induced SH-SY5Y neuroblastoma cell differentiation. **(A)** Nucleoside transporter blockers (10 µM NBTI plus 100 µM propentofylline–PPF-plus 10 µM dypiridamole -DIP-) were simultaneously added to the medium containing 1% serum 1 h before GUO (100 µM) treatment and until the end of the experiment (48 h), while the antagonist of A_1_ and A_2A_ adenosine receptor, DPCPX (100 nM) and ZM241385 (50 nM), respectively, were added 30 min before and until the end of the 48 h GUO (100 µM) treatment period. **(B)** The selective inhibitors of PI3K (25 µM LY294002), PKC (1 µM GF109203X), and sGC (10 µM OQD) or **(C)** the selective inhibitors of NOS (5 μM L-NAME), HO (1 µM ZnPP) and sGC (10 µM OQD) were added to cultures before the addition of GUO (100 µM) and during all the GUO treatment. In all cases, after 48 h, the total number of neurite-bearing cells was determined. Each value represents the mean ± SEM of at least five independent experiments in duplicate and is expressed as a percentage of neurite-bearing cells vs. untreated cells. Statistical analysis was performed using one-way ANOVA with the Dunnett’s or Turkey’s post-hoc multiple comparisons test; *p < 0.05, **p < 0.02, ****p < 0.001 compared with untreated cells; #*p* < 0.05, # #*p* < 0.02, # # #*p* < 0.01 compared with GUO treated cells.

To evaluate if the GUO differentiating effect was due to its extracellular activity, as previously reported for some effects (Giuliani et al., 2012a; Giuliani et al., 2015), nucleoside transporter blockers (10 µM NBTI plus 100 µM propentofylline-PPF- and 10 µM dipyridamole-DYP-) were added 1h before and during GUO treatment (100 μM, 48 h). The uptake inhibitors produced only a partial reduction of about 20% of the GUO-induced effect (Figure 3A), without affecting untreated cell differentiation when administered alone (data not shown). Since GUO effects might be due to adenosine receptor activation (Dal-Cim et al., 2012), we next assessed adenosine receptors involvement by adding selective antagonists for A_1_ (DPCPX, 100 nM), or A_2A_ receptors (ZM241385, 50 nM) to cell cultures treated with GUO 100 µM for 48 h. Neither DPCPX nor ZM241385 altered the ability of GUO to enhance SH-SY5Y cell differentiation (Figure 3A), as well as neither by themselves, affected the behavior of untreated cells (data not shown).

Finally, we investigated some possible signaling pathways involved in GUO-induced neuron-like cell differentiation. As known, pathways such as that of PI3K, PKC, and cGMP play a key role in many physiological functions including cell differentiation (Heikkilä et al., 1993; Kimura et al., 1994; Bau et al., 2005). Thus, we analyzed their participation in the GUO effect, adding selective inhibitors of PKC (GF1094002, 1 µM), or PI3K (LY294002, 25 µM), or sGC (ODQ, 10 µM) to the culture medium treated with GUO 100 µM for 48 h. The PKC and sGC inhibitors reduced GUO-induced differentiation by about 24 and 31% respectively, while the PI3K inhibitor did not affect it (Figure 3B). Since either HO-1 or NOS can stimulate sGC (Cary and Marletta, 2001), to further investigate the upstream pathways involved in cGMP formation, cells were exposed, to L-NAME (5 µM) or ZnPP (1 µM), the selective inhibitors of NOS or HO activity, respectively. As shown in Figure 3C, the HO inhibitor, but not the NOS inhibitor, significantly reduced the GUO effect on neuron-like cell differentiation.

### Exogenous Guanosine Added to SH-SY5Y Cell Cultures was Metabolized to Guanine

**FIGURE 4:**
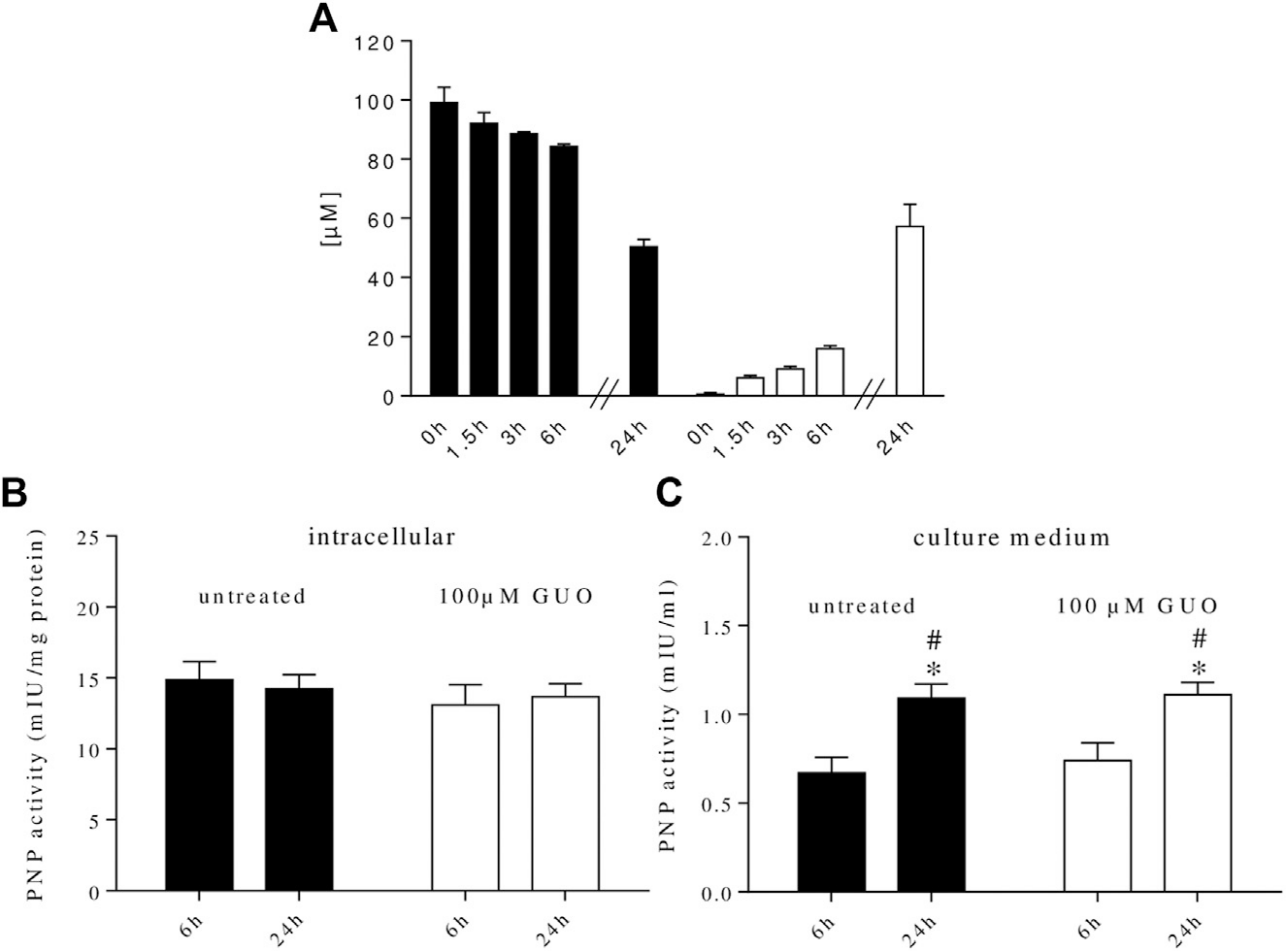
Metabolic fate of extracellular guanosine and purine nucleoside phosphorylase (PNP) activity in SH-SY5Y cell cultures. **(A)** GUO (100 µM) was added to the culture medium containing 0% serum. At the indicated times, an aliquot of the medium was taken and analyzed by HPLC as described in materials and methods. **(B)** PNP activity was measured within SH-SY5Y neuroblastoma cells or **(C)** in the culture medium. For this purpose, SH-SY5Y cells were incubated in a serum-free medium supplemented or not with 100 µM GUO. After 6 and 24 h, an aliquot of the medium was taken and the enzyme present was concentrated using Amicon Ultra filters while cells were scraped in lysis buffer and cytosolic extracts were prepared. PNP activity was assayed using 100 μM GUO as substrate plus 50 mM Pi as co-substrate for 15 min at 37°C. The concentration of the newly formed product, GUA, was measured by HPLC analysis. PNP activity was expressed as milli-International Units (mIU) per mg protein or ml of culture medium. Values are the mean ± SEM of at least five independent experiments, each run in duplicate. Statistical analysis was performed using a two-way ANOVA followed by Turkey’s multiple comparison test, *p < 0.05 compared with untreated cells at 6 h and #*p* < 0.05 compared with GUO treated cells at 6 h.

It is known that GUO is intracellularly metabolized to GUA by PNP enzyme. We recently found that PNP is also present in human plasma (Giuliani et al., 2016) and that rat C6 glioma cells, astrocytes, and microglial cells (Giuliani et al., 2017; Peña-Altamira et al., 2018), can release PNP in the extracellular milieu. Thus, to better analyze the impact of GUO on cell differentiation, we examined the metabolic fate of GUO in our cultures. By HPLC analysis, we found that GUO (100 µM) added to the cell cultures was no longer present extracellularly after 48 h, while the concentration of GUA, its direct metabolite, was 112 ± 18 µM. To avoid interference with serum, in which PNP is present, a serum-free medium was used and cell cultures were treated with 100 µM GUO for 24 h, a time period in which cell viability was not modified by serum absence (Supplementary Figure S1). In this condition, exogenous GUO levels decreased over time, while increasing concentrations of GUA concurrently appeared in the medium (Figure 4A).

We also evaluated PNP activity inside and outside cells. At the two time periods considered (6 and 24 h), in both untreated and GUO-treated cells, the intracellular PNP activity was constant over time (Figure 4B) and was always higher than the extracellular one (Figure 4C). On the contrary, outside the cells, the enzyme activity was higher after 24 h either in untreated and treated cells (Figure 4C), indicating that these cells were able to release PNP that accumulated in the extracellular milieu. Interestingly, GUO treatment was not effective in modulating PNP release since PNP activity was not modified vs. untreated cells (Figure 4C). When added to the reaction mixture, the PNP inhibitor, forodesine 1 μM, prevented the formation of GUA, thus confirming the specificity of the enzymatic assay.

## Discussion

The treatment of patients with high-risk NB remains a challenge since even after surgical resection, chemo- and radiotherapy, relapse is common and can depend on the presence of poorly differentiated cells (Whittle et al., 2017). For this reason, differentiating agents are used in the attempt to eradicate minimal residual disease, thus improving the clinical outcome. Even if some differentiating agents have been identified, only RA is used but, unfortunately, resistance to this treatment is frequent (Whittle et al., 2017), highlighting the need to identify new potential agents. Among purine compounds, GUO may behave as a potential differentiating agent; thus, this study was designed to evaluate its neuritogenic effect on NB cells. Here, we have chosen the SH-SY5Y cells since it is a human-derived NB cell line and this should avoid species differences in the interpretation of data. Furthermore, since serum present in the culture medium contains many substances that can affect cell differentiation (Brunner et al., 2010) and its composition can vary from one lot to the next, to reduce serum interferences and experimental variations, we used a culture medium containing the lowest concentration of serum that did not affect cell survival, that is, 1% FBS, as the most suitable experimental condition.

Upon these experimental conditions, we showed that GUO was effective in inducing SH-SY5Y cell differentiation in a time- and concentration-dependent manner, as revealed by the increased number of neurite-bearing cells. Furthermore, the changes in cell morphology and neurite length were also associated with an increased presence of mature neuronal markers indicating their differentiation into neuronal-like cells. Although several studies have shown that GUO promotes cell differentiation of different cell types (Di Liberto et al., 2016), here we presented further new data for SH-SY5Y cells. Noteworthy, GUO was effective in inducing differentiation of PC12 cells and B16F10 melanoma cells (Gysbers and Rathbone, 1996; Bau et al., 2005; Naliwaiko et al., 2008) that, like SH-SY5Y NB cells, share a common neural crest origin. Furthermore, in melanoma cells, GUO strongly decreased cell motility, thereby contributing to reduce tumor malignancy. GUO has also been evaluated as a potential therapeutic agent in several types of neoplasms such as lung cancer and hepatoma (Yang et al., 2005; Su et al., 2010) and it has been shown to increase the antitumor effect of chemotherapeutic agents such as 5′-deoxyfluorouridine, acriflavine, and temozolomide (Iigo et al., 1987; Kim et al., 1997; Oliveira et al., 2017). Furthermore, while chemotherapeutic agents can upregulate several pro-metastatic and pro-survival factors and are associated with high toxicity to non-tumor cells (Ratajczak et al., 2013), there is no evidence of serious adverse effects after systemic administration of GUO (Schmidt et al., 2010; Jackson and Mi, 2014). Overall, these considerations suggest GUO as an interesting potential antitumor agent to be used, maybe, in combination with other conventional agents in a multimodal fashion.

After having established that GUO stimulated neuron-like cell differentiation, we next investigated the possible mechanism of action and the signaling pathways behind this effect. Importantly, GUO-mediated neuritogenesis was not abolished when its uptake was blocked by nucleoside transporter inhibitors, thus indicating that this effect was mainly exerted by extracellular GUO. These data agree with our previous findings (Giuliani et al., 2012a; Giuliani et al., 2015) confirming that, besides displaying several intracellular roles, GUO can be considered an extracellular signaling molecule. However, while specific GUO binding sites have been found on rat brain membranes (Traversa et al., 2002; Traversa et al., 2003; Volpini et al., 2011; Frinchi et al., 2020), the identity of these membrane proteins have not yet been unequivocally identified. Interestingly, an indirect mechanism has been proposed by which GUO exerts its effect *via* adenosine receptor-mediated signaling, in particular A_1_, A_2A_ receptors, and A_1_/A_2A_ receptor heteromers (Dal-Cim et al., 2012; Lanznaster et al., 2019). However, in our hands, neither the A_1_ nor the A_2A_ selective receptor antagonists modified the GUO-mediated differentiation effect. These results corroborate the hypothesis that GUO might act through putative specific sites distinct from adenosine A_1_ and A_2A_ receptors.

Many diseases, such as cancers, require treatment with a combination of drugs to obtain superior effects or to prevent the emergence of resistance. Therefore, the identification of molecular mechanisms involved in GUO-induced differentiation is of fundamental relevance since combination of drugs that target different cellular pathways may work synergistically to cell killing. Starting from the evidence that GUO activated some signaling pathways including PI3K, PKC, and sGC (Bau et al., 2005; Nailiwaiko et al., 2008; Dal-Cim et al., 2012; Giuliani et al., 2015), which have long been recognized to be also involved in neurite outgrowth (Heikkila et al., 1993; Kimura et al., 1994; Bau et al., 2005), here we tested the activity of selective inhibitors of those signals on GUO-mediated neurite outgrowth. While the PI3K pathway was not involved, PKC and sGC signaling pathways are required for the GUO effect since their inhibition strongly attenuated differentiation. However, since neither PKC nor sGC inhibitor completely abolished GUO-effect, we believe that GUO-induced differentiation requires the activation of further signal transductions, such as a cyclic adenosine monophosphate pathway, as reported in other cells (Gysbers and Rathbone, 1996). This aspect needs to be still investigated in SH-SY5Y cells.

Noteworthy, since GUO signal is also linked to the activation of enzymes such as HO or NOS, leading to increased production of CO or NO, respectively, which in turn stimulate sGC to generate cGMP (Cary and Marletta, 2001; Cary et al., 2006; Ryter et al., 2006), we investigated whether HO or NOS were involved in the GUO-induced effect. Our data demonstrated that HO or sGC inhibitors decreased the GUO effect while the NOS inhibitor was ineffective. These data corroborate those observed in PC12 cells (Bau et al., 2005) and strengthen the importance of the HO/sGC pathway in GUO-induced differentiation.

Finally, we could not overlook that cell purine homeostasis is ensured by a complex network of enzymes, localized both intra- and extracellularly, and by membrane transporters, and that enzymes controlling purine metabolism play a key role in regulating the biological effects of extracellular purines (Volonté and D’Ambrosi, 2009). Thus, since GUO was added to the culture medium for a long time, we studied its metabolic fate in the extracellular medium. Indeed, after 48 h exogenously administered, GUO was no longer present in the culture medium, whereas we detected only GUA. Exogenous GUO could be taken up into the cells, transformed into GUA, and then released outside the cells. Indeed, we found a strong PNP activity inside SH-SY5Y cells. Nonetheless, there is also the possibility that GUO could be metabolized extracellularly. Unlike ectonucleotidases (that convert nucleotides into nucleosides), the presence of extracellular enzymes metabolizing purine nucleosides and nucleobases is still a matter of debate. However, since no GUO kinase exists in mammals (Ipata, 2001), the first step in the metabolism of exogenous GUO should be its transformation into GUA by extracellular PNP. Noteworthy, the PNP presence in the culture medium was not due to cell death since cell viability was not modified as evaluated by MTT test, and PNP activity increased along time without modification upon GUO treatment. Overall, these data corroborate those already found in rat glioma C6 cells and in rat astrocytes and microglial cells (Giuliani et al., 2017; Peña-Altamira et al., 2018) and further support the existence of a constitutive release of PNP from cells that, in these experiments, was unaffected by GUO treatment. Data on GUO metabolic fate raise some questions. Indeed, recent studies highlighted biological effects also for GUA (Giuliani et al., 2012b; Zuccarini et al., 2018), although some signaling pathways involved in the GUO pro-differentiating effect are peculiar of GUO rather than GUA (e.g., HO and PKC) (Zuccarini et al., 2018). In this perspective, it is noteworthy that Garozzo et al. (2010) found that GUA, more than GUO, can exert antiproliferation effects in human glioma cell lines; however, this effect was mainly due to an intracellular effect, while in the present study, the GUO effect was mainly extracellularly mediated since nucleoside transporter inhibitors did not abolish it. The evaluation of a potential GUA role in NB cell differentiation is currently being tested, and some preliminary pilot experiments conducted using forodesine, to inhibit the degradation of exogenous added GUO, did not seem to modify GUO-induced differentiation, but further experiments will be addressed to unravel the interplay between GUO, PNP, and GUA to identify new therapeutic targets.

In conclusion, findings from this study demonstrate that GUO is effective in inducing NB cell differentiation, activating a process in which some molecular mechanisms have in part been identified (such as PKC, HO, and sGC cascades). Indeed, these results open a new perspective for NB treatment, thus further investigation on the role of GUA and the functioning of the complex guanine-based purine signaling in NB cell differentiation might yield relevant implications for NB therapeutic purposes.

## Data Availability

The datasets presented in this article are not readily available because the raw data supporting the conclusions of the article will be made available by the authors, upon reasonable request, to any qualified researcher. Requests to access the datasets should be directed to Patricia Giuliani, patricia.giuliani@unich.it.
