# Supplementary figures and images for: Investigating the Role of Guanosine on Human Neuroblastoma Cell Differentiation and the Underlying Molecular Mechanisms
Source: Front Pharmacol. 2021 Apr 27;12:658806. doi: 10.3389/fphar.2021.658806 (PMC8111303; doi:10.3389/fphar.2021.658806)

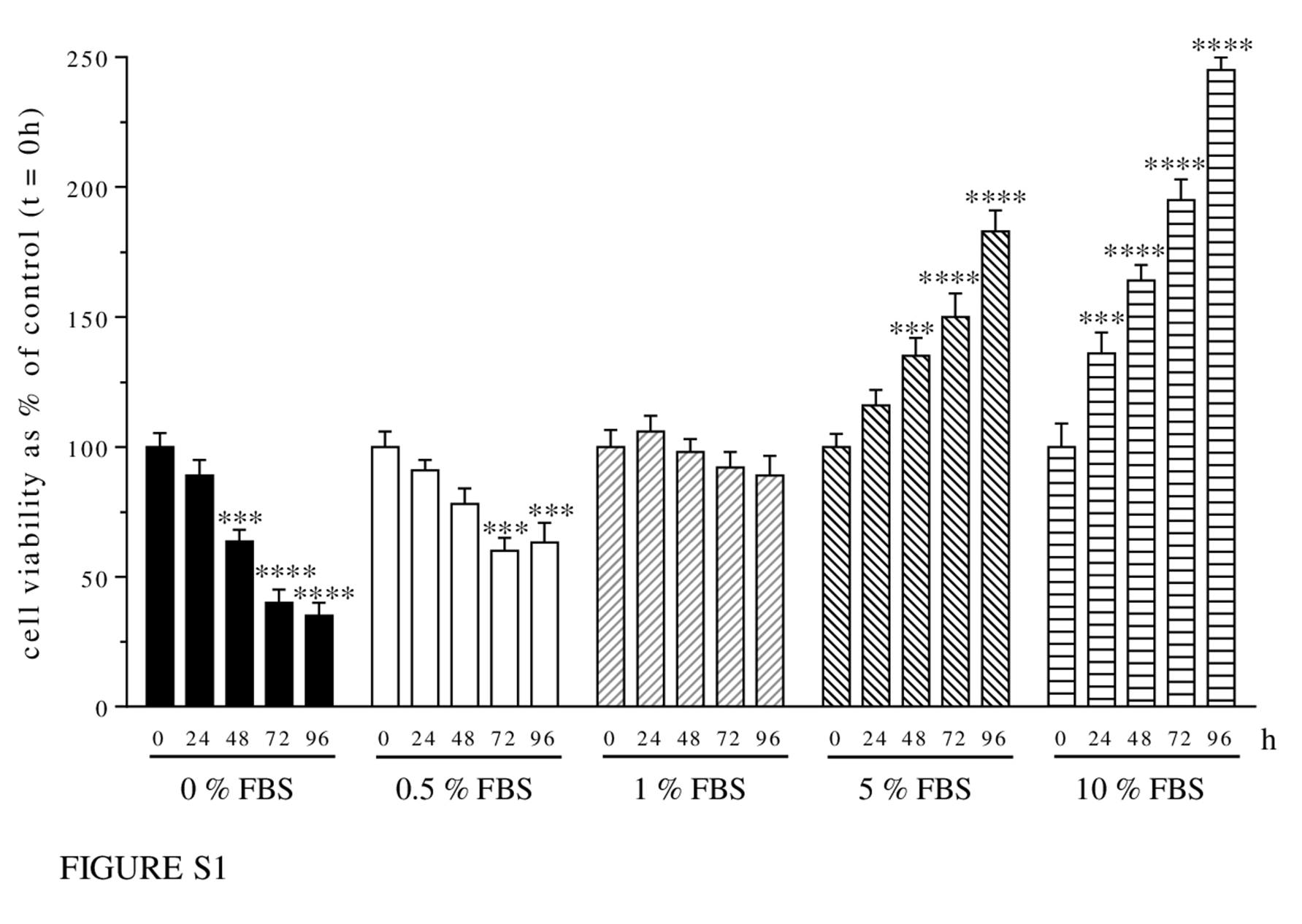

Supplement: Supplementary file 1 [file image1.tiff]

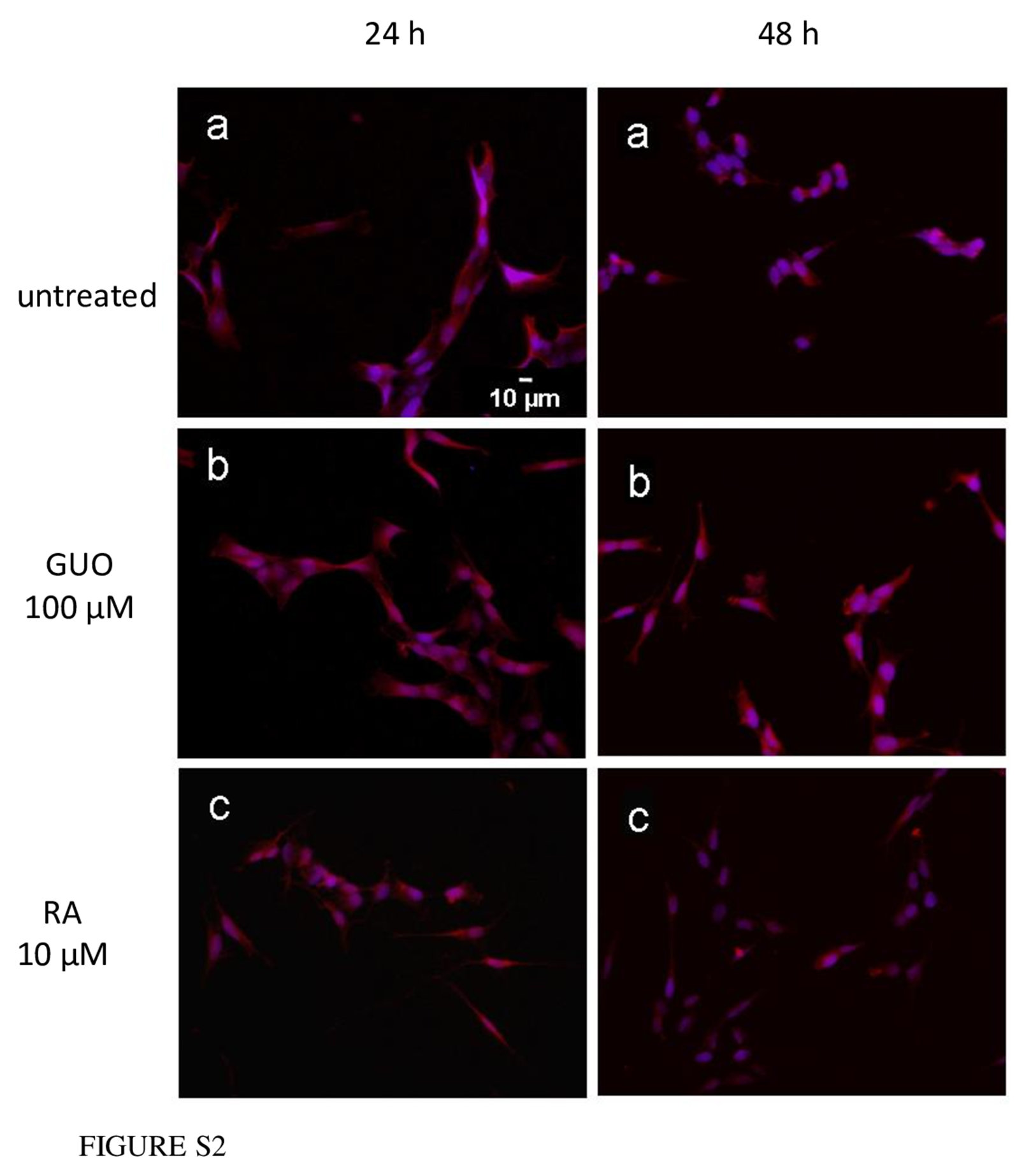

Supplement: Supplementary file 3 [file image2.tiff]
