# Supplementary material for: Investigating the Role of Guanosine on Human Neuroblastoma Cell Differentiation and the Underlying Molecular Mechanisms
Source: Front Pharmacol. 2021 Apr 27;12:658806. doi: 10.3389/fphar.2021.658806 (PMC8111303; doi:10.3389/fphar.2021.658806)
Supplement: Supplementary file 2 [file Presentation1.pdf]

Frontiers in Pharmacology – Supplementary Information

Belluardo et al.

Supplemental Figures

FIGURE S1 Relationship between serum concentrations in the medium and SH-SY5Y cell survival. SH-SY5Y cells were seeded on 96-well plates ($3x10^3$ cells/well) and cultured for different times in a medium without serum or containing increasing serum concentrations. Cell viability was evaluated using the 3-[4,5-dimethylthiazol-2-yl]-2,5-diphenyltetrazolium bromide (MTT) assay. At the end of each experiment, MTT was added to each well to a final concentration of 0.5 mg/ml and maintained for 2h at 37°C in a tissue culture incubator. Then the medium was removed, dimethyl sulfoxide was added to dissolve the neoformed formazan, and the absorbance was quantified spectrophotometrically at a wavelength of 550 nm (Microplate Autoreader, Bio-Tek instruments). Values represent a percentage of the respective control (t = 0h) and are expressed as mean ± SEM from at least three to five independent experiments performed in triplicate. Statistical analysis was performed using two-way ANOVA with Dunnett's post-hoc multiple comparisons test; ***$p<0.01$, ****$p<0.001$ compared with respective control (t = 0).

FIGURE S2 Effect of guanosine and retinoic acid on phalloidin expression. Since actin filaments reorganization play an important role in the definition of neuronal morphology during differentiation, long-term changes in the cytoskeleton arrangement was monitored using phalloidin staining. Phalloidin binds specifically to F-actin, and fluorescent-tagged phalloidin stains the actin skeleton in cells in a manner that is very close to the staining pattern seen using anti-actin antibody (Wulf et al., 1979). SH-SY5Y cells were cultured for 24 and 48 h without treatment (a) or in presence of 100 µM GUO (b) or 10 µM RA (c). Cells were then fixed with 2 % paraformaldehyde for 30 min, washed with phosphate-buffered saline (PBS) and permeabilized in 0.1 % Triton-X-100 in PBS for 5 min. Then, cells were incubated in a phalloidin solution diluted 1:1000 in PBS for 15 min. After incubation, cells were washed with PBS and then mounted on coverslips with DABCO-Glycerol. F-actin filaments are stained in red while nuclei are stained in blue with DAPI. Images are representative of one of three independent experiments.

Reference

Wulf, E., Deboben, A., Bautz, F.A., Faulstich, H., Wieland, T. (1979) Fluorescent phallotoxin, a tool for the visualization of cellular actin. Proc Natl Acad Sci USA 76 (9), 4498-4502.
